# Open by default: a proposed copyright license and waiver agreement for open access research and data in peer-reviewed journals

**DOI:** 10.1186/1756-0500-5-494

**Published:** 2012-09-07

**Authors:** Iain Hrynaszkiewicz, Matthew J Cockerill

**Affiliations:** 1 BioMed Central Ltd, 236 Gray’s Inn Road, London, WC1X 8HB, UK

## Abstract

Copyright and licensing of scientific data, internationally, are complex and present legal barriers to data sharing, integration and reuse, and therefore restrict the most efficient transfer and discovery of scientific knowledge. Much data are included within scientific journal articles, their published tables, additional files (supplementary material) and reference lists. However, these data are usually published under licenses which are not appropriate for data. Creative Commons CC0 is an appropriate and increasingly accepted method for dedicating data to the public domain, to enable data reuse with the minimum of restrictions. BioMed Central is committed to working towards implementation of open data-compliant licensing in its publications. Here we detail a protocol for implementing a combined Creative Commons Attribution license (for copyrightable material) and Creative Commons CC0 waiver (for data) agreement for content published in peer-reviewed open access journals. We explain the differences between legal requirements for attribution in copyright, and cultural requirements in scholarship for giving individuals credit for their work through citation. We argue that publishing data in scientific journals under CC0 will have numerous benefits for individuals and society, and yet will have minimal implications for authors and minimal impact on current publishing and research workflows. We provide practical examples and definitions of data types, such as XML and tabular data, and specific secondary use cases for published data, including text mining, reproducible research, and open bibliography. We believe this proposed change to the current copyright and licensing structure in science publishing will help clarify what users – people and machines – of the published literature can do, legally, with journal articles and make research using the published literature more efficient. We further believe this model could be adopted across multiple publishers, and invite comment on this article from all stakeholders in scientific research.

## Introduction

Much has been written about, and support stated for, sharing and publishing scientific data, in recognition of the benefits for the economy [1], scientific discovery [2] and public health [3]. Maximizing the potential of scientific data sharing for the discovery of new knowledge involves reducing barriers to data dissemination, reuse, reproducibility and integration. Licensing, ownership, copyright and intellectual property present legal obstacles to data integration and reuse, which has led to the development of, and calls for, licensing standards for open data; where data are explicitly placed in the public domain with legal rights of the owners waived [4].

BioMed Central has previously stated that the concept of open data, analogous to its policy on open access to journals, goes beyond making data freely accessible. Data should also be free to distribute, copy, re-format, and integrate into new research, without legal impediments [5]. This position is consistent with the Panton Principles, which hold that for society to reap the full benefits of scientific research the published body of knowledge must be open – readily available such that it can be evaluated, reused, criticized and integrated with other knowledge without restrictions [6]. For the remainder of this article the term ‘open data’ is reserved exclusively for data available according to these principles.

Unfortunately much data – and other content – freely available on the web are available under restrictive or ambiguous terms, which risks impeding or potentially criminalizing secondary users of scientific data. According to evidence submitted to the UK Government’s intellectual property review by the Wellcome Trust, 87 per cent of the material housed in the full-text scholarly archive UK PubMed Central is unavailable for legal text and data mining [7]. A key finding of a more recent report, commissioned by JISC, was a need to overcome legal restrictions and uncertainties surrounding text mining of scientific literature [8].

Indeed, as recognition of the value of shared life science data has increased, so has recognition of intellectual property and copyright as barriers to progress. Writing in *Nature* in 2009, Schofield *et al*., urged that “any restrictions on use should be strongly resisted and we endorse explicit encouragement of open sharing” [9]; and Conway and VanLare in JAMA, in 2010, called for US health care data to be available without intellectual property constraints [10]. Waiver of all intellectual property rights in research data is central to the achievement of an “information commons”, advocated by organisations such as Sage Bionetworks, to enhance the (slowing) pace of drug discovery.

The genomics community has shown leadership in establishing a framework for an “information commons”, engrained in the Bermuda Principles, and have established built-in temporal latencies to data for knowledge (when data are released), and rights (when rights restricting use are removed) [11]. Researchers in this community typically must release their genetic sequence data immediately, and within 6–12 months release their exclusive rights in that data. During this relatively short embargo researchers have their opportunity to exploit the data for their discoveries, after which the community at large can benefit, if they wish, from the new data. A similar model for data release has since been proposed for clinical trials, although is probably far from implementation [12]. A number of factors seem to have led to a successful culture of sharing in the genomics community: a need to collaborate and share to achieve a major goal (the sequencing of the human genome); effective mechanisms and infrastructure for sharing large amounts of data (well-funded genetic sequence databases); scientific community and funding agency mandates to share data; and importantly, in the context of this article, successful collaborations with the publishing community. Journals, their editors and publishers, supported implementation of the Bermuda Principles by, for example, requiring accession number for data deposits as a condition of manuscript submission or publication.

BioMed Central in its August 2010 open data statement [5] and subsequent cross-publisher Publishing Open Data Working Group meeting identified that open data in journal publications could be implemented by specifying that, from a specific date, any author submitting to a journal or publisher agrees to dedicate the data elements of their article and supplementary material (in particular, additional data files; also known as “supplementary” data files) to the public domain [13]. Much of the contents of academic journals could be considered as data but licensing terms cannot be applied retroactively by publishers without authors’ consent, and any changes to authors’ agreements should ideally be made in consultation between authors and publishers.

This article aims to describe practically what is needed from publishers to explicitly dedicate data within open access journals to the public domain, and discusses the implications of this development for authors, editors, publishers and funders of research. Illustrative examples and use cases are provided throughout the article. In this article “open access” is defined according to the Budapest Open Access Initiative definition [14].

## Applying the right license to published research and data

The internet has revolutionized the way we access and distribute information, enabling virtually anyone to post content online. There is much potential in rapidly sharing content on the web, but releasing content without information, or with ambiguous information, about if and how it can be shared and reused can also cause problems – especially for data.

Open access publishing of peer-reviewed journal articles commonly utilizes the legal tools – licenses – prepared by Creative Commons. BioMed Central, Public Library of Science, Nature Publishing Group, BMJ and many others publish open access articles where the authors retain the copyright to their work. Authors typically apply a Creative Commons attribution license (CC-BY), or variation of it, which means anyone is free to copy, reuse, distribute and make derivatives from their article provided that there is attribution of the original author(s). However, many “open access” publishers place restrictions on commercial reuse of published articles (papers) and on creation of derivative works, which can include text mining in some jurisdictions. Additionally, some commercial publishers’ terms and conditions, by contract, can prevent text mining in any jurisdiction. Commercial use restrictions have been strongly discouraged – their use described as amounting to “pseudo open access” – as authors will not reap the full benefits of paying for open access publication (for example figures could not be uploaded to Wikipedia with commercial use restrictions) [15,16]. BioMed Central supports unrestricted use of open access content including commercial use and as such requires authors to apply a CC-BY license by default. BioMed Central’s full text corpus of open access research articles published under CC-BY is available for free distribution, reuse and creation of derivatives with no commercial use restrictions – with data mining research strongly encouraged [17]. For data published by scholarly publishers, the Association of Learned and Professional Society Publishers and International Association of Scientific, Technical, & Medical Publishers (STM) issued a joint statement in 2006 supporting sharing of raw datasets among scholars and recommending that publishers do not require transfer of copyright in data submitted for publication [18].

### Copyright and data

The policies and guidelines of many academic institutions advise researchers to establish intellectual property and copyrights at the start of any project (although whether the issue of data ownership is consistently addressed by researchers is unclear [19]). Copyright cannot generally be asserted in facts, only the ways in which they are presented. At a basic level raw data are merely simple, mathematical, descriptions of facts and to claim copyright a scientist would need to exert individual judgment, expression or skill in their representation. For example, Einstein could not claim copyright in the formula E = mc^2^, but could in text explaining the theory behind it [20]. You could conclude from this that copyright and associated licenses and attribution requirements cannot legally be applied to data. However, there are many levels at which data – particularly digital data derived and integrated from different sources – and collections of data and metadata can operate and be represented, and many ways in which copyright law is applied in different jurisdictions.

In the US the law focuses on creativity (“Copyright does not protect facts, ideas, systems, or methods of operation, although it may protect the way these things are expressed”) but in Australia originality is more important – and copyright may well apply to research data “in the same way that it applies to written works like books, journal articles and reports” [21]. In the European Union “*sui generis*” rights exist to protect data within digital databases – effectively, copyright – which can, furthermore, be implemented differently by member states. Because of these substantial international legal differences regarding how copyright can be applied to data, there are inherent difficulties in ascertaining the extent of copyright in a dataset. A more comprehensive summary of the different approaches to copyright in data and databases can be found in [22]. All of these issues compound the uncertainty about what an individual or machine (such as a computer crawling the web) can do, legally, with information they download from the internet, including from journals.

### Licenses and waivers for data

A license is a legal instrument for a copyright holder or content producer to enable a second party to use their content, and apply certain conditions and restrictions to those uses. A waiver is also a legal instrument but is designed for a rights holder to *give up* their rights, rather than assert them. For a comprehensive guide to the different approaches to the licensing of research data see [23].

Placing restrictions on the reuse of scientific information, particularly data, slows down the pace of research. Furthermore, legal requirements for attribution ingrained in licenses such as CC-BY can prohibit future research across large collections of content – as commonly happens in data mining research. Consider the Human Genome Project: a watershed moment for scientific data sharing and collaboration. Without the collective effort of many different research institutions, commercial organizations and individual scientists the sequencing of the human genome would not have been possible. But if a researcher wishing to query the human genome database as part of a new research project was legally required to attribute all the – probably thousands – of data contributors, by providing a link back to or citation, this would be unmanageable, and probably un-publishable in the context of a traditional research paper’s reference list.

International legal differences, described earlier, are another important reason to apply specific, appropriate legal tools to data. Also, it can be unclear what license to attach to copyright in a dataset or structure (for example a textual description of building the dataset could fall under CC-BY, but if source code were used rather than text it might not). This is an area of confusion where no licensing standard exists. Therefore, to eliminate legal impediments to integration and re-use of data, such as this stacking of attribution requirements in large collections of data, and to help enable long-term interoperability an appropriate license or waiver specific to data should be applied. There are a number of conformant licenses and waivers for open data [24], of which Creative Commons CC0 (http://creativecommons.org/publicdomain/zero/1.0/) is widely recognized. Under CC0, authors waive all of their rights to the work worldwide under copyright law and all related or neighboring legal rights they have in the work, to the extent allowable by law. Legal experts have recommended the use of standard, globally accepted licenses for data instead of developing *ad hoc* models [25].

### The case for CC0 for scientific data

The Creative Commons’ website catalogues a number of different organizations – publicly and privately funded – which use CC0 for data [26]. These include:

• Genomes Unzipped, which “aims to inform the public about genetics via the independent analysis of open genetic data, volunteered by a core group of genetics researchers and specialists”

• GlaxoSmithKline (GSK), a leading pharmaceutical company, has dedicated data on more than 13,500 compounds known to be active against malaria to the public domain [27].

• The British Library and Cologne-based Libraries, which have released large amounts of bibliographic data under CC0 [28]

• FigShare (http://figshare.com/), a freely-accessible repository for scientific content including images, video and data, uses CC0 for datasets

Data repositories are particularly relevant users of waivers and licenses for research data. Although there are many data repositories in life sciences (for a list see http://www.datacite.org/repolist), which are growing in size and number, not all scientific domains have a common repository and journals often function as repositories when data are included as additional files (supplementary material). Dryad (http://datadryad.org/) is an international repository for the datasets supporting published, peer-reviewed journal articles across the biosciences which requires authors to explicitly place deposited data in the public domain using the CC0 waiver. An entry on the Dryad weblog sets out cogently why CC0 is the most effective solution for achieving its goals:

“*By removing unenforceable legal barriers, CC0 facilitates the discovery, re-use, and citation of [that] data…*

“*Furthermore, Dryad’s use of CC0 to make the terms of reuse explicit has some important advantages:*

• ***interoperability:****Since CC0 is both human and machine-readable, other people and indexing services will automatically be able to determine the terms of use.*

• ***universality:****CC0 is a single mechanism that is both global and universal, covering all data and all countries. It is also widely recognized.*

• ***simplicity:****there is no need for humans to make, and respond to, individual data requests, and no need for click-through agreements. This allows more scientists to spend their time doing science*.” [29]

Dryad’s policy ultimately follows the Science Commons’ recommendations, set out in their Protocol for Implementing Open Access Data [30].

The online laboratory notebook software LabArchives (http://www.labarchives.com/), which includes the ability to share data privately and to publish datasets publicly and permanently online, also uses CC0 for public datasets [31].

## Concerns about public domain dedication of data

### Credit where credit’s due – attribution and citation

A common concern about moving from an attribution license, such as CC-BY, to CC0 for data and waiving attribution rights is that academic credit (citations) will be lost if there is no longer a legal requirement to attribute the original rights holder (author). While attribution can sometimes be achieved in the same way as citation the two practices serve different purposes. Attribution is a legal tool designed to permit copying, distribution, and creation of derivative works such as translations. As copyright does not protect ideas (in the US), to give scientists credit for their ideas the established norm in scholarly communication is citation [32].

Consider a scientist paraphrasing a concept put forward in a peer’s research article. He or she does not legally have to cite their peer’s published paper, but it is beneficial or possibly essential for the validity and reliability of the subsequent work to specify the source(s) of assertions made. Community norms are enforced by the community, and in science unacceptable citation practices are typically identified and resolved through peer review, and the publication ethics and editorial policies of peer-reviewed journals. See Table 1 for common citation and attribution events in scholarly communication. These examples are for illustrative purposes and do not constitute legal advice.

**Table 1:** Attribution vs. citation in (re)uses of open access scientific content published under a Creative Commons Attribution license (CC-BY)

**Activity**	**Attribution and/or citation?**	**Explanation**
Printing an article for display at a conference	Attribution	Printing an article is redistribution so covered by copyright (and attribution is achieved inherently by the authors’ names and copyright ownership being stated on the article)
Translating article for publication in another journal	Attribution + citation	Attribution is required as a translation is a derivative work, and most journal duplicate publication policies (an ethical requirement) require citation of the original paper for republications
Paraphrasing a concept or finding within a paper	Citation	If you rely on another scientists idea for your work credit is due to the previous author through citation
Reusing a figure, table or graph	Attribution + citation	Reusing a figure, table or graph is copying and redistribution, so requires attribution; by presenting another scientist’s representation of their data you need to give credit to their original work
Publication of a reanalysis of data published as an additional file in a journal	Citation	The source of the data being reanalyzed may not legally need to be attributed if copyright does not apply (e.g. in the US), even if the data are included with the secondary publication, but for the reanalysis to stand up to scrutiny – and pass peer review – the source of the data must be cited

The examples in Table 1 demonstrate that, although they can sometimes be achieved in the same way, attribution and citation are not the same. Citations are much more important and relevant than attribution when tracking scholarly outputs and giving appropriate credit for individuals’ contributions.

Compared to legal requirements, cultural norms benefit from flexibility, and can evolve with the community which established them. In other words, using norms retains control and decision making within the research community, instead of taking it out of our control and handing it to lawyers and judges. Many scientific ideas, after a number of years, become undisputed and the community may deem it unnecessary for credit to be rigorously applied. For example, it would today be very unusual for an article describing a DNA sequencing experiment to cite the original work by Watson and Crick that elicited DNA’s structural properties. This is a cultural norm at work, where an idea is now so widely accepted, and the initial authors clearly recognized for their discoveries (in citations and prizes) a citation is not needed.

Jonathan Rees, formerly of the Creative Commons, said of community norms for influencing behavior over legal requirements: “*For widest latitude of use and best scalability, and therefore greatest return to the research community, the entirety of the data set, including any incidental copyrightable elements, should be dedicated to the public domain. Note that public domain is not incompatible with a request for attribution or other terms of use following community norms. Such a request may be as effective – or more effective – at getting users to follow desired practices as any attempted legal restrictions *[33].”

To our knowledge there have been no empirical studies of the citation of scholarly datasets assigned public domain dedication licenses compared to a comparable group available under attribution licenses. However, given public domain dedication – and specifically CC0 – is intended to maximize the potential for data discovery, reuse and therefore citation, it would be reasonable to hypothesize that citation potential of public domain data would be unaffected or might even increase. With CC0 there is no need for transfer agreements and preconditions, which inherently impede further (re)uses of data. Sharing of microarray experimental research data underlying journal articles has been associated with increased citation share [34], and increased reproducibility and repeatability of results [35]. In social science data collections funded by the National Institutes of Health and National Science Foundation in the US, data sharing has been associated with “many more times the publications” than collections where data were not shared [36]. Linking of publications to supporting datasets has also been associated with more citations to the linked paper in the marine science journal *Paleoceanography*, according to a conference abstract, and in the field of astronomy according to a pre-print paper [37].

### Competition

Researchers who apply CC0 to their data, or any other product of their scholarship, waive all rights in that data allowable by law. Such a waiver has been described as an “unattractive option for data whose creators have yet to fully exploit them, academically or commercially” [23]. This is true, but a waiver or license required by a journal or publisher generally applies only in the context of data submitted for publication. If a portion of a large database was analyzed and an additional data file included for publication the larger, unpublished, body of work would retain whichever license the researchers, their employers or institutions require. In other words, researchers remain in control of what they chose to publish – what they submit to a journal – and a change in the publishers’ license does not affect this. Moreover, waivers and licenses for journal articles do not replace existing, established community norms for sharing of some data types (e.g. depositing microarray and genetic sequence data in appropriate databases) – nor do they affect requirements of many journals for sharing readily reproducible materials including raw data on request [38].

There is a trade-off between the additional opportunities which may result from transparency (such as new collaborations, secondary use) and the threat, improbable or otherwise, that opening up data may be valuable to competitors. Certain types of research, such as genetic sequencing to elucidate susceptibility to disease, generates far more data than one research team could conceivably analyze – which logically lead to sharing and collaboration. A number of companies have opened up some of their data and seen benefits [39]. A lot of data may have commercial value but much raw data, such as protein sequences of potential drug targets, are just the beginning of a knowledge-discovery process. More can be gained by “pre-competitive” sharing with the waiver of intellectual property. Such an approach is being championed by Sage Bionetworks in the US [40].

### Plagiarism

Plagiarism is research misconduct and an unfortunate but ineliminable occurrence in scholarship. Plagiarism and the potential for plagiarism have increased with the proliferation of digital access to information [41]. Plagiarism is often not illegal, but it is certainly unethical, and undoubtedly damaging for the career of someone guilty of perpetrating plagiarism. Effective online tools for detecting plagiarism exist, such as CrossCheck (http://www.crossref.org/crosscheck/index.html), as does human detection via the peer-review process. Removal of a legal requirement for attribution for data elements of articles would be unlikely to impact on the potential for plagiarism. In addition, CC0 would not apply to the main, copyrightable text of articles.

### Other safeguards

Public domain dedication of data does not mean that those who generated the data cannot express certain wishes about how the data are used. The Panton Principles frequently asked questions (FAQs) state: “*You should always aim to follow any reasonable requests made by the data owners/publishers. These may be explicit or may be implicitly understood by the community. You should make an effort to understand any relevant ‘community norms’ for the data you are using *[42]*.”*

A code of conduct has been proposed for those wishing to reuse clinical trial data obtained from other researchers [43] and a clinical trial dataset published in the journal *Trials*, by Sandercock *et al*., has requested that “any publications arising from the use of this dataset acknowledges the source of the dataset, its funding and the collaborative group that collected the data.” [44].

Electronic publishing platforms provide further safeguards to ownership and authorship of published content, in the form of date stamping manuscript submissions and version control in some repositories, such as Edinburgh DataShare (http://datashare.is.ed.ac.uk/).

Citation of articles and datasets is facilitated through standard citation formats – such as those advocated by DataCite where persistent dataset identifiers, such as digital object identifier (DOI) names, are displayed as linkable, permanent URLs – and are increasingly supported by some publishers [37].

## What do we mean by data?

There are numerous definitions of data. According to Wikipedia data “are qualitative or quantitative attributes of a variable or set of variables. Data are typically the results of measurements and can be the basis of graphs, images, or observations of a set of variables,” [45]. Data can exist electronically or non-electronically, so a definition that includes electronic access is important, in the context of integration, reuse and data mining of online scholarly content. The Copyright, Designs and Patents Act, part of UK statute, uses a broad definition of databases incorporating electronic access:

“*Databases(1)In this Part “database” means a collection of independent works, data or other materials which—*

(a)are arranged in a systematic or methodical way, and

(b)are individually accessible by electronic or other means.

*(2)For the purposes of this Part a literary work consisting of a database is original if, and only if, by reason of the selection or arrangement of the contents of the database the database constitutes the author’s own intellectual creation*.”

Other definitions, such as at the United States National Science Foundation ‘DataNet’ program, have been broader and implied anything capable of existing digitally, including publications and software, could be considered data [46]. The former definition is more broadly applicable to data which can be harvested, mined or downloaded from open access journals – and to which CC0 rather than CC-BY should apply. This inevitably means information which can be processed by machines as well as being transferred electronically by them (e.g. papers attached to emails) [47]. It is not possible to comprehensively define and account for all data and data file types, particularly given the rapidly evolving nature of data and text mining applications, but a number of general examples and definitions follow below. We strongly encourage readers to comment on these data definitions and provide additions and amendments. These examples intentionally do not include domain-specific data standards (agreed upon formats for disseminating and presenting particular types of scientific experiments), which are comprehensively catalogued by BioSharing (http://biosharing.org/?q=standards).

### Tabular data

Data elements organized in columns and rows – a table – are extremely common in scientific publications. While attribution would be required for reproduction in whole or in part of a table as presented in a journal publication, the individual values and collection of values should be considered as data and therefore open. Data, in the course of a scientific experiment, are usually collected at a greater level of detail than are reported in a paper, with tables reporting summary or mean values. Although these data are aggregated from the raw data they remain numerical representations of a fact, and therefore data. Tables are furthermore often included as additional files in a variety of formats including PDF, HTML/XML, DOC and Excel/CSV. Ideally all tables included in the main body of a journal article should also be included as additional CSV files – an open, machine-readable format – but when they are not present as tables in journal articles CSV would represent good practice for tabular data. Proprietary file types, such as Microsoft Office, and formats which are not readily editable, such as PDF, are not recommended for tabular data provided as additional data files.

### Graphs and graphical points

Graphs, graphical representations of relationships between variables, are ostensibly images and therefore not, when considered as a collective entity, data. However, the individual data points underlying a graph, similar to tables, certainly are. An example of best practice when submitting a manuscript with a graph to a peer-reviewed journal would be for authors to also submit accompanying CSV tables with the corresponding data points, so that graphs could be re-plotted. Although this practice is required by some specific journals it is not widespread. However, software tools exist that are capable of “scraping” underlying data points from graphs and images (for example http://www.chardta.com/) and can be useful, for example, for enhancing the discoverability of scientific information by exposing underlying data points to internet search engines.

### XML

According to the World Wide Web Consortium (W3C), “Extensible Markup Language, abbreviated XML, describes a class of data objects called XML documents and partially describes the behavior of computer programs which process them” [48]. XML is widely used as a standard for data transfer and for creating versions of works intended for machine reading, and therefore to be used as data. Therefore for our purposes we can assume XML files are data. XML has many applications in science and is frequently published with journal articles as additional files in BioMed Central journals as well as underlying the online articles themselves. XML forms the basis of many domain-specific data standards such as Gating-ML in flow cytometry, FuGE-ML in functional genomics, GelML in gel electrophoresis and so on (see: http://biosharing.org/standards_view).

### Bibliographic data

Bibliographic data have been historically described as information not included in the full text and images included with an article, which includes reference lists. “Core bibliographic data” have been further described as “data which is necessary to identify and / or discover a publication” and defined under the Open Bibliography Principles:

• names and identifiers of author(s) and editor(s)

• titles

• publisher information

• publication date and place

• identification of parent work (*e.g.* a journal)

• page information

• Uniform resource identifiers (URIs) [49]

Therefore, these core bibliographic data should be considered open data.

### RDF

Resource Description Framework (RDF) is a standard language for encoding data and metadata on the web. It is designed to indicate the relationship between online objects in a human and machine-readable way, and facilitate merging of data between different sources even if the underlying schemas of the sources are different. RDF forms the basis of the semantic web, and is a core component of achieving Tim Berners-Lee’s vision of Linked Data on the web [50]. RDF provides new opportunities for data and knowledge management in life sciences, chemistry, and publishing. All BioMed Central journal articles, for example, contain embedded RDF, which conveys harvestable information about content, such as authors, licensing information and the unique identifier for the article [51].

### What aren’t data?

Although source code may be represented as data and is certainly machine readable there are a wide range of existing licensing systems and community norms that exist around software. Therefore we choose to regard software, compiled code, and source code as a separate category and not as data. Specific licenses and repositories have been developed for source code for software and Open Source Initiative compliant licenses [52] are recommended. Files pertaining to programming languages can be included as additional files with journal publications, either directly in formats such as SQL or indirectly in compressed or packaged file formats such as ZIP.

There are myriad file types which can be published as additional files but amongst the most common, in BioMed Central journals, are those usually pertaining to text and written works – PDF and DOC/DOCX and HTML (a full list of published additional data files is available on request from BioMed Central [53]). Caution is recommended in the interpretation of these objects as open data.

## Implementing a variable license for open access research and data

### Setting date (CC) Zero

Creative Commons licenses (CC-BY, specifically) have provided an effective and penetrative solution for digital copyright in open access scientific works (papers). But as the nature of the published scientific paper (article) has evolved then so too should the copyright and licensing structure which authors apply to their content. Published articles are increasingly collections of different digital objects, perhaps including a few thousand words of text, half a dozen images and a similar number of CSV or XML (data) files.

The fact that CC-BY is a suboptimal license for data does not mean that the many thousands of published authors have done something wrong, as CC-BY was (and often still is) the best instrument available when copyright license agreements for open access journals were prepared (although, CC-BY version 4.0, currently in draft form, aims to tackle the issue of *sui generis* database rights [54], described earlier). A number of data repositories, including Dryad and FigShare, initially asked authors to make their deposited data available under a Creative Commons attribution license but have since changed their policy (https://twitter.com/#!/figshare/statuses/50241486796754944). However, licenses and waivers cannot be applied retroactively by a publisher without explicit consent of the copyright holder(s) – in the vast majority of cases at BioMed Central, the authors. A small number of datasets remain in Dryad which are not available under CC0, as explicit agreement from data depositors (rights holders) could not be obtained to change the terms of data release [29].

A change to BioMed Central’s standard license agreement to include a CC0 waiver for published data would remove ambiguities about the copyright and attribution requirement status of parts of published articles and associated data files, and enable instead the application of scientific, cultural norms that meet the needs of scientists better than an inflexible legal instrument [33,55]. To implement open data in journal publications the new license agreement would need apply to all authors from a specific date, such that any author submitting to a journal/publisher agrees to dedicate the data elements of their article and additional files to the public domain. A proposal for how this could be reflected in published articles’ copyright license statement follows:

"© 2012 <Author> et al. This is an Open Access article distributed under the terms of the Creative Commons Attribution License (http://creativecommons.org/licenses/by/2.0), which permits unrestricted use, distribution, and reproduction in any medium, provided the original work is properly cited. Data included in this article, its reference list(s) and its additional files, are distributed under the terms of the Creative Commons Public Domain Dedication waiver (http://creativecommons.org/publicdomain/zero/1.0/; http://www.biomedcentral.com/about/access).”

This would be further indicated in article metadata, RDF and on the journal and publisher’s policy pages and author submission pages online. This addition to the statement aims to succinctly summarize that data in published articles generally originates from three sources – tabular data or text-minable factual data (e.g. numerical instances of a particular word or phrase, such as gene/protein names) in the main body of an article; additional (supplementary) files such as XML and CSV file extensions that include data; and the reference list (bibliographic data). From a legal perspective, we might need to be more comprehensive about our definitions of data (see ‘What do we mean by data?’, above) in the full legal code of a new license and in guidelines accompanying this proposed change. And practically, authors would need to agree to apply two different legal tools to content they submit for publication as part of the submission process – with links to both CC-BY and CC0, for example, in journal submission checklists.

The need for change in author behavior, as a result of this proposed modification, is minimal. CC0 would not apply to nor affect the availability of data not submitted for publication; we would instead be asking authors to apply different terms of use to a proportion of the content they already publish. By submitting a manuscript and all associated files to a BioMed Central journal authors already confirm that they agree to all terms of the BioMed Central Copyright and License Agreement [56], including that they are able to apply a CC-BY license. This proposed change to the license agreement should provide clarity to the licensing of specific components of published articles and does not represent a substantial change to the overall license agreement for authors’ published work.

### Open data by default – opt out

Public domain dedication of data, while universally desirable, is not always universally possible. Authors, for the most part, prepare research articles in the context of employment by a third party and can be subject to licensing terms which supersede the standard terms of a publisher. This already happens for a small proportion of the content published by BioMed Central and undoubtedly by other publishers, such as some articles funded by the World Health Organization and US government. Therefore, any submitting author who is not able to agree to all terms of the BioMed Central Copyright and License Agreement should contact the journal editorial office at the earliest opportunity – ideally before, during or immediately after manuscript submission. The onus is on the authors and, if applicable, their employers to decide on the applicability of the publisher’s standard license agreement to their work and whether an exception is needed. Alternatives to the standard license agreement can be discussed. This process of checking suitability of the standard license agreement, and requesting alternatives where necessary, therefore already happens for all manuscripts submissions. The owners of the rights (if any) in the data must decide if their data can be made open. According to the Panton Principles FAQs “[i]n most cases, the people who make a decision to publish, and were intimately involved in the generation of the data, should be making this decision.” [42]

### Publishing platform developments

Implementing a new license agreement will have technical as well as policy and procedural implications. While it is not possible to specify these changes in detail here, and is beyond the scope of this document, the following would be essential for implementation:

• Tagging of articles and data files published under a non-standard license agreement (where authors have opted out of the new default open access-open data license)

• Editing standard embedded license information in article XML metadata and RDF and a tool to automate insertion of non-standard licensing terms

• Insertion of license information to additional files and associated metadata

Furthermore, the following would be desirable to enhance the discoverability and usefulness of open data in journal articles:

• Tagging and classification of published data files, for example by file type

• A tool to automatically discover and aggregate additional files

• A tool to (retrospectively) associate data objects with papers on the web

• Approaches to associating published datasets with journal articles which go beyond hyper-linking, such as through linked data methods

• Searching within and filtering of additional files

## Open data in science use cases in published and unpublished contexts

There are many uses for open data but probably many more as yet unknown. As stated by Tim Berners-Lee and Nigel Shadbolt in *The Times* on New Year’s Eve 2011, “*One reason that the worldwide web worked was because people reused each other’s content in ways never imagined or achieved by those who created it. The same will be true of open data.*”

The examples that follow focus on licensing and reuse of data included with and/or harvestable from journal publications, in the context of the proposed change to BioMed Central’s standard license agreement, above, for open access and open data journal articles.

### Example #1 – analysis of a large clinical trial dataset

In April 2011 Sandercock *et al*., published in *Trials* ‘The International Stroke Trial Database’ [44], which “aimed to make individual patient data from the International Stroke Trial (IST), one of the largest randomised trials ever conducted in acute stroke, available for public use, to facilitate the planning of future trials and to permit additional secondary analyses.”

The “database”, including 19,000 anonymized individual patient data, is available with the journal article as a 4.3 Mb CSV file (http://www.trialsjournal.com/content/supplementary/1745-6215-12-101-s1.csv) under a CC-BY license. With the new license agreement as proposed in this article the CSV file would be available for reuse without a legal requirement for attribution engrained in CC-BY. Secondary uses for this dataset might include a novel secondary analysis by a different group of researchers, and analysis and integration of the dataset in the context of a systematic review and meta-analysis of randomized trials of heparin and/or aspirin in acute ischemic stroke. Both of these activities might conceivably result in further publications. Although there would be no legal requirement for attribution, for any secondary article about this dataset – or indeed any systematic review – to be scientifically valid it would need to cite its source(s) of data.

### Example #2 – Application of magnetic resonance techniques to cross-species comparative studies

Magnetic Resonance Imaging (MRI) techniques are used to better understand the evolution of specific traits in animals and cross-species comparisons (for example in primates) are particularly important. But due to ethical, practical and funding limitations single studies typically are only able to consider one or two species. There is only one publicly available dataset that has brains from multiple primate species scanned according to a common protocol and these scans (of 11 species) were recorded (in vivo) well over a decade ago, and so do not meet the quality criteria that underpin more recent brain morphometric algorithms of the kind required for cross-species studies of brain structure. However, a review of this area of research found that “the major barrier to cross-species MR-based brain morphometry is not the lack of data nor analytical tools but barriers preventing to combine them” [57]. Open data in this field would undoubtedly drive new discoveries.

### Example #3 – Research utilizing text and data from journal publications

The copyright status of data obtained though text-mining is debatable. The numerical instances of a particular gene or protein name in a full-text corpus of articles could be valuable for secondary research and, in the US at least, are likely to be considered (non-copyrightable) facts. Some scholars take the position that mining does not violate copyright law because it does not meet the statutory definition of copying which requires “fixing” the work in a permanent form [15]. Yet text mining is often restricted by commercial publishers. In the study of small angle scattering (a “technique based on the deflection of a beam of particles, or an electromagnetic or acoustic wave, away from the straight trajectory after it interacts with structures that are much larger than the wavelength of the radiation” according to Wikipedia [58]), a researcher might be interested to harvest the data used in other publications to test their analysis tools and provide better teaching aids. Generally, in this area of research, the data are only presented as a graph, the data analysis is not spelt out and there is no specific license attached if the data are available (Cameron Neylon, personal communication).

### Example #4 – Open bibliography

Online scientific publishing has driven a diversification of measures of research and researcher impact, extending the focus from journal impact factors to article and individually-led metrics. Bibliographic information (rather than copyright attribution), which enables identification of scholarly work and tracks citations to scientists’ work, is central to earning of academic credit for concepts and ideas. Many services are now available which enable individual authors to calculate their citation index, known as the Hirsch or h-index. Examples include Scopus, Thomson ISI, Google Scholar and Microsoft Academic Search. However, much of the data underlying these metrics is not available openly, leading to multiple scores for the same individual or paper – depending on the tool or service used, which have different corpuses and different algorithms for calculating impact scores. A common, open bibliography, as has been established by some leading libraries would enable anyone to assess, utilize and build applications based on the data [59]. And furthermore, from a researcher’s perspective this approach is far more efficient, negating the need to maintain and report multiple sources of data from multiple impact-measuring tools. As outlined by Jones *et al.*[49] the motivations for and opportunities for open bibliographies are many. The negative implications of open bibliography for an author of a paper are negligible. Under the license agreement proposed in this article CC0 would apply to the article title, author names and information, unique identifying and publishers’ information, and reference list. Given a primary use of bibliographic information is to track scholarly citation activity, authors could reasonably expect these open data to increase the visibility and impact of their work.

### Example #5 – Reproduction/validation of results for teaching and further research

In September 2010 Tommi Nyman and colleagues published an article in *BMC Evolutionary Biology*, “How common is ecological speciation in plant-feeding insects? A 'Higher' Nematinae perspective” [60]. The article included, in addition to the sequence data used to reconstruct the phylogenetic trees, the background data used in the phylogeny-based ecological analyses as additional file 1 – an Excel file. The data are well labeled and readily understandable by other scientists and fully document how they sampled their insects. This informative approach means, for example, readers would not need work through the references to discover the sampling used. These data have potential usage for reproduction and validation of the article’s findings, for teaching purposes, and conceivably uses involving the processing and integration of the data using computer software. Explicit dedication of these data to the public domain minimizes barriers to these scientifically important activities and maximizes the reuse potential of the data, as we could be more confident that all future uses of the data will not be impeded by licensing restrictions.

## Concluding remarks – and what next?

Legal issues present substantial barriers, in theory and reality, to the reuse and integration of research data which are free to access online, and data published in peer-reviewed journals. The implementation of a new license and waiver agreement, as per the protocol described in this article, in BioMed Central journals and in the future by other open access publishers should help further realize the benefits of open data for the scientific community – and beyond. We invite all our readers and authors to consider and comment on the implications of the proposed change to BioMed Central’s license agreement set out in this article.

## Competing interests

MC is Managing Director of BioMed Central, part of Springer Science + Business Media. IH is an employee of BioMed Central.

## Authors' contributions

The idea for this manuscript was conceived at a meeting (reported on the BioMed Central Blog, [13]) which was attended by MC and a number of those listed in the acknowledgements section of this article. The meeting was chaired by IH. IH wrote the first draft of the manuscript. MC was involved in critical review and editing of the original manuscript, and revised versions thereof, in response to comments received from the acknowledged contributors. Both authors read and approved the final manuscript.
